# Causal relationship between immune cells and Guillain-Barré syndrome: a Mendelian randomization study

**DOI:** 10.3389/fneur.2024.1446472

**Published:** 2024-11-12

**Authors:** Huaiquan Liu, Shuoshuo Shao, Bo Chen, Shili Yang, Xinyan Zhang

**Affiliations:** Guizhou University of Traditional Chinese Medicine, Guiyang, Guizhou, China

**Keywords:** Mendelian randomization, genome-wide association study, immune cells, Guillain-Barré syndrome, causal relationship

## Abstract

**Objective:**

The aim of this study was to investigate the causal effect of immune cell phenotype on GBS using two-sample Mendelian randomization (MR) approach.

**Methods:**

This study used MR to investigate the causal relationship between 731 immune cell phenotypes and GBS. We used Inverse variance weighted, Weighted median, MR Egger, Simple mode, Weighted mode for MR analysis. We also used the Cochran Q test, MR-Egger intercept test, IVW regression and MR-PRESSO, leave-one-out analysis to assess the presence of horizontal pleiotropy, heterogeneity and stability, respectively.

**Results:**

Our study revealed a causal relationship between 33 immune cell phenotypes and GBS. Twenty immunophenotypes were observed to be associated with GBS as risk factors. For example, CD20 on IgD+ CD38dim in the B cell group (OR = 1.313, 95%CI:1.042–1.654, *p* = 0.021), CD3 on CD4 Treg in Treg cell group (OR = 1.395, 95%CI:1.069–1.819, *p* = 0.014), CD3 on TD CD8br in Maturation stages of T cell group (OR = 1.486, 95%CI:1.025–2.154, *p* = 0.037), CD16 on CD14+ CD16+ monocyte in Monocyte group (OR = 1.285, 95%CI:1.018–1.621, *p* = 0.035), CD33dim HLA DR+ CD11b + %CD33dim HLA DR+ in Myeloid cell group (OR = 1.262, 95%CI:1.020–1.561, *p* = 0.032), HLA DR+ NK AC in TBNK cell group (OR = 1.568, 95%CI:1.100–2.237, *p* = 0.013). Thirteen immune phenotypes are associated with GBS as protective factors. For example, CD19 on PB/PC in the B cell group (OR = 0.577, 95%CI:0.370–0.902, *p* = 0.016), CD4 Treg AC in Treg cell group (OR = 0.727, 95%CI:0.538–0.983, *p* = 0.038), CD11c + monocyte %monocyte in cDC group (OR = 0.704, 95%CI:0.514–0.966, *p* = 0.030), CX3CR1 on CD14+ CD16− monocyte in Monocyte group (OR = 0.717, 95%CI:0.548–0.939, *p* = 0.016), Mo MDSC AC in Myeloid cell group (OR = 0.763, 95%CI:0.619–0.939, *p* = 0.011), CD45 on granulocyte in TBNK group (OR = 0.621, 95%CI:0.391–0.984, *p* = 0.042).

**Conclusion:**

The findings suggest that certain specific immune cell phenotypes, particularly B cell and Treg cell subpopulations, are causally associated with GBS, providing potential targets for the clinical treatment of GBS.

## 1 Introduction

Guillain-Barré syndrome (GBS) is a peripheral neurological disorder that mainly involves nerve roots and peripheral nerves, and is usually triggered by infections, such as intestinal or respiratory infections (1). The onset of GBS is usually rapid, with rapid progression within hours to days, peaking within 2–4 weeks, and is characterized by symmetrical movement disorders. Typical signs and symptoms include weakness or paralysis of the limbs, sensory deficits, loss of reflexes, pain, autonomic dysfunction, facial paralysis, dysphagia and respiratory distress (2). Most patients with GBS partially improve or recover with treatment, while a few may still have prolonged weakness or other problems (3, 4). Epidemiological studies have shown that the global annual incidence of GBS is approximately 1–2/100,000 population, with high mortality and disability rates, and that it occurs in males and in people over 50 years of age (5, 6).

Unfortunately, the exact pathogenesis of GBS is not yet fully understood and is usually considered to be closely related to factors such as respiratory or intestinal infections, recent immunizations and autoimmune diseases. These factors may induce an abnormal immune system response, causing the immune system to mistakenly attack the peripheral nerves, which in turn destroys the myelin sheath. It is noteworthy that GBS is clinically categorized into demyelinating and axonal forms, namely, acute motor axonal neuropathy antibody-mediated (AIDP) and acute motor sensory axonal neuropathy (AMAN). Among them, anti-ganglioside antibodies, particularly anti-GM1 antibodies, are deeply related to the pathogenesis of AMAN. By contrast, the relationship between AIDP and autoantibodies has not been fully clarified, whereas the phagocytosis of myelin by macrophages is a well-known pathological feature in AIDP (7). In the initial stage of GBS, a large number of lymphocytes and macrophages can be seen infiltrating around the lesion nerve, and after activation, a large number of inflammatory cytokines can be produced, resulting in demyelination and axonal damage (8). Immunotherapy regimens such as plasmapheresis (PE) and intravenous immunoglobulin (IVIG) are considered to be one of the key therapeutic options in the treatment of GBS (9–11). In the field of tumor immunotherapy, GBS is one of the adverse events of concern in immune checkpoint inhibitors (ICIs) (12). Each of these mechanisms involves a complex immune response, and it is urgent to explore the causal relationship between immune cells and GBS as soon as possible (13, 14).

Currently, studies surrounding the association between GBS and immune cells are insufficient. And the direct causal relationship between them is highly susceptible to the confounding factors of clinical research and becomes elusive. Therefore, this study took advantage of the fact that genetic variants are randomly assigned to individuals before birth, and conducted a two-sample MR analysis using Genome-wide association study (GWAS) data to further search for a causal relationship between immune cell causal relationship with GBS.

## 2 Materials and methods

### 2.1 Study design

**Figure 1:**
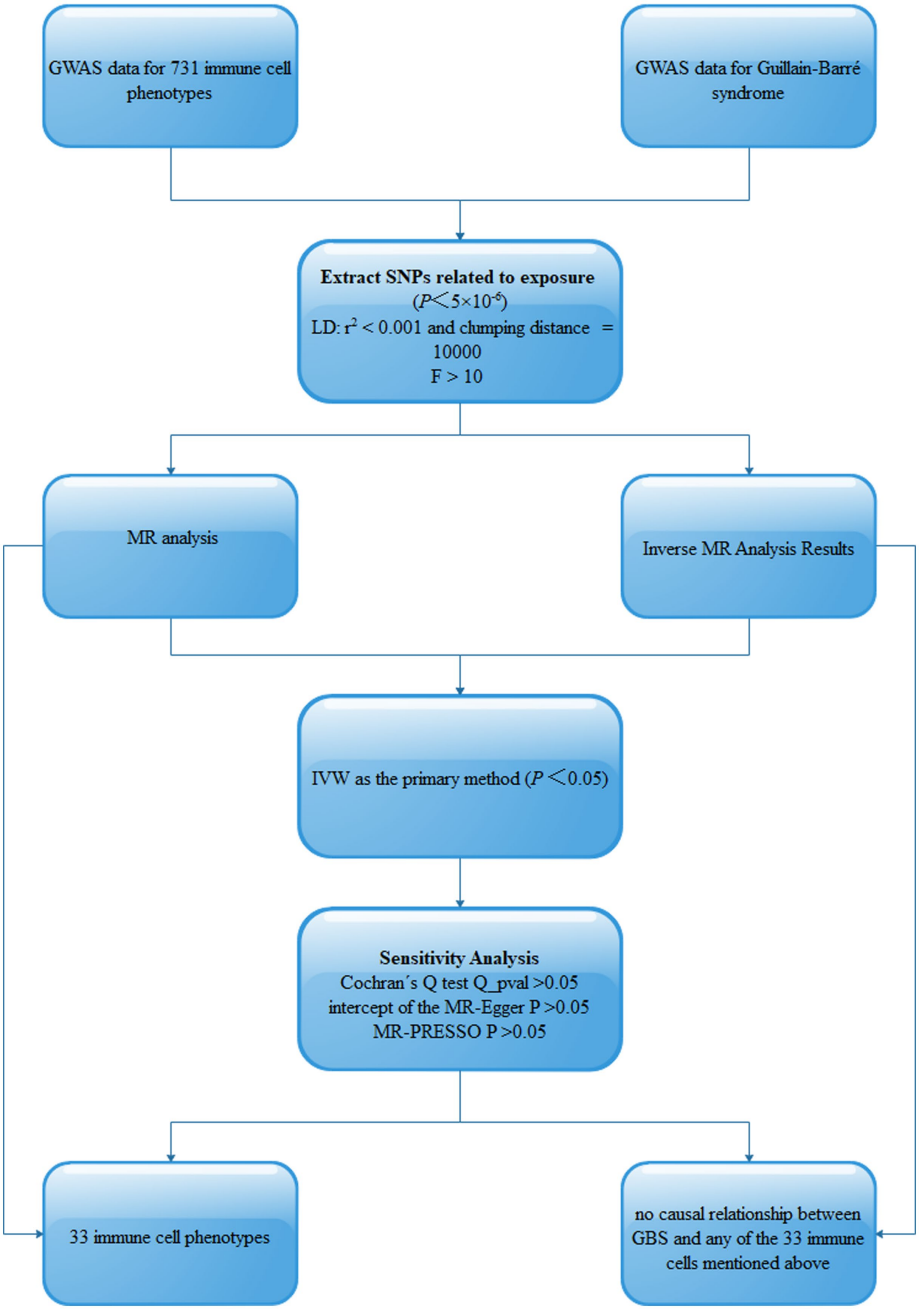
Schematic of MR analysis of 731 immune cell phenotypes causally associated with GBS. SNPs, single-nucleotide polymorphisms; Linkage disequilibrium (LD), Mendelian randomization (MR), Inverse variance weighted (IVW).

This study assessed the potential causal relationship between 731 immune cell phenotypes and GBS using two-sample MR analysis. This study should be guided by three basic assumptions: (1) genetic variation is directly related to exposure; (2) there are no potential confounders between genetic variation and exposure and outcome; and (3) genetic variation does not affect outcome through pathways other than exposure. The comprehensive design of this work is shown in Figure 1.

### 2.2 Data source for the GWAS

The GWAS data for 731 immune cell phenotypes and GBS were obtained from the IEU OPENGWAS database, which is publicly available (15). The 731 immune cell phenotypic data contained four immunomorphological features, namely absolute cell count (AC, *n* = 118), relative cell count (RC, *n* = 192), median fluorescence intensity (MFI, *n* = 389), and morphological parameters (MP, *n* = 32). Specifically, they are subdivided into seven major groups: B cell, T regulatory cells (Treg), classical dendritic cells (cDC), Lymphocyte subsets (TBNK), Maturation stages of T cell, Myeloid cell and Monocyte. Specific information is provided in Table 1.

**Table 1:** Summary information on GWAS data.

GWAS data	Sample size	Population	Data sources	SNP(n)	Year
Immune cells	3,757	European	ebi-a-GCST0001391- ebi-a-GCST0002121	About 2,000,000	2020
Guillain-Barré syndrome	215,931	European	finn-b-G6_GUILBAR	16,380,463	2021

### 2.3 Instrumental variables selection

Suitable Instrumental Variables (IVs) were obtained separately from different datasets for MR analysis. For IVs with immune cell characteristics associated with GBS we set the *p*-value threshold to 5 × 10^−6^. We set the parameters to *r*^2^ < 0.1 and kb = 10,000 for linkage disequilibrium (LD) analyses, and excluded the effect of confounding factors. To prevent alleles from influencing the results, palindromic SNPs were removed by palindromic sequence detection. *F* > 10 were included for MR analysis to exclude bias in weak IVs.

### 2.4 Statistical analysis

In this study, five complementary methods were used for MR analysis: Inverse variance weighted (IVW), Weighted median, MR Egger, Simple mode, Weighted mode. IVW as the primary method of analysis, with *p* < 0.05 considered statistically significant and in the same direction as the results of the other methods of analysis, we then considered a causal relationship between exposure and outcome. We checked the heterogeneity of the IVs using the Cochran Q test, with Q_pval>0.05 indicating no heterogeneity. The MR-Egger intercept test, Mendelian randomization pleiotropy residual sum and outlier (MR-PRESSO) test were used to detect horizontal multiplicity and outliers, setting *p* < 0.05. Robustness and symmetry were further assessed by leave-one-out analysis to test whether individual SNPs contributed to the causal effect. All statistical analyses in this study were performed using the R package “TwoSampleMR.”

### 2.5 Reverse MR analysis

We investigated whether immune cell characteristics are affected by GBS using inverse MR analysis, further validating the directionality of the causal effect. We performed a reverse MR analysis with GBS as the exposure factor and the above immune cell profile as the outcome to further validate the directionality of the causal effect of immune cell profile and GBS.

## 3 Results

### 3.1 Causal relationship between immune cells and GBS

**Figure 2:**
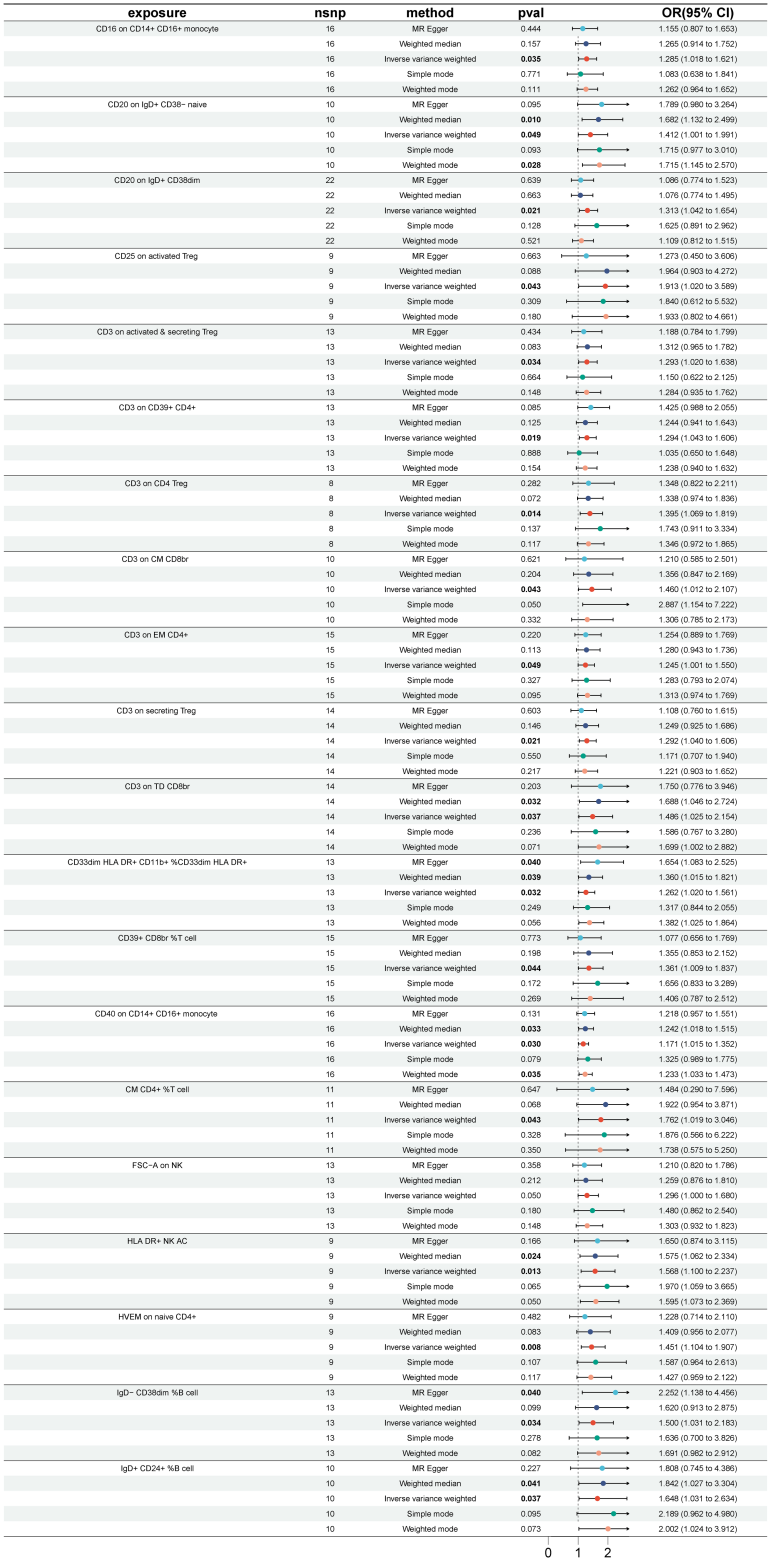
Forest plot for the causal effect of 20 immune cell features as risk factors for GBS using 5 methods including MR Egger, Weighted median, Inverse variance weighted, Simple mode, Weighted mode.

**Figure 3:**
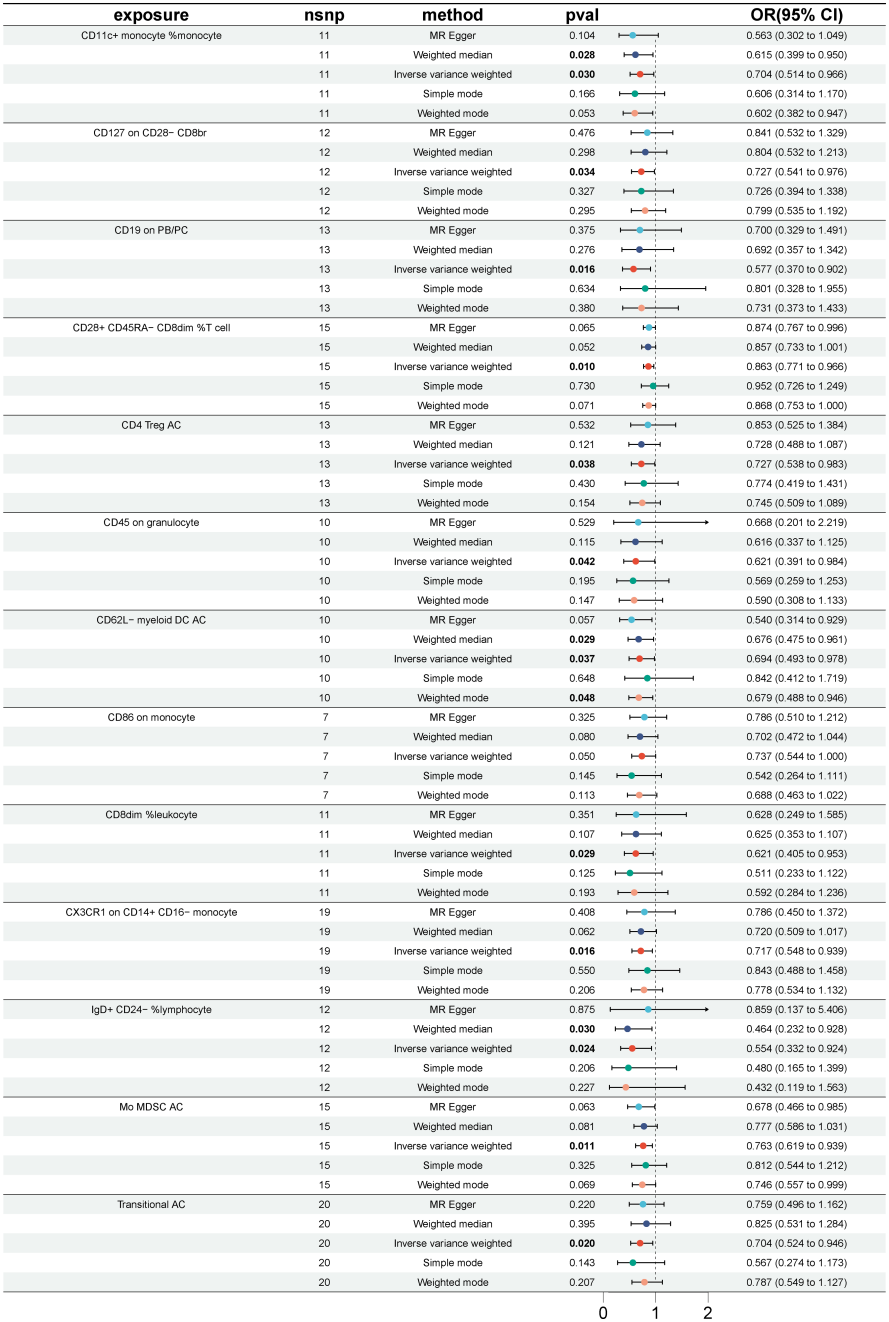
Forest plot for the causal effect of 13 immune cell characteristics as protective factors for GBS using 5 methods including MR Egger, Weighted median, Inverse variance weighted, Simple mode, Weighted mode.

A total of 33 immune cell phenotypes were identified by the IVW method as being causally associated with the development of GBS, setting *p* < 0.05. The 33 immune cell types included 20 risk factors and 13 protective factors. There were 9 cases in the Treg cell group, 7 cases in the B cell group, 5 cases in the Maturation stages of T cell group, 4 cases in the TBNK group, 3 cases in the cDC cell group, 3 cases in the Monocyte group and 2 cases in the Myeloid cell group. Twenty immunophenotypes were found to be associated with GBS as risk factors using the IVW method. For example, in the B cell group, CD20 on IgD+ CD38− naïve (OR = 1.412, 95%CI:1.001–1.991, *p* = 0.049), CD20 on IgD+ CD38dim (OR = 1.313, 95%CI:1.042–1.654, *p* = 0.021), IgD− CD38dim %B cell (OR = 1.500, 95%CI:1.031–2.183, *p* = 0.034), IgD+ CD24+ %B cell (OR = 1.648, 95%CI:1.031–2.634, *p* = 0.037). In the Treg cell group, CD25 on activated Treg (OR = 1.913, 95%CI:1.020–3.589, *p* = 0.043), CD3 on activated and secreting Treg (OR = 1.293, 95%CI:1.020–1.638, *p* = 0.034), CD3 on CD39+ CD4+ (OR = 1.294, 95%CI:1.043–1.606, *p* = 0.019), CD3 on CD4 Treg (OR = 1.395, 95%CI:1.069–1.819, *p* = 0.014), CD3 on secreting Treg (OR = 1.292, 95%CI:1.040–1.606, *p* = 0.021), CD39+ CD8br %T cell (OR = 1.361, 95%CI:1.009–1.837, *p* = 0.044). In the Maturation stages of T cell group, CD3 on CM CD8br (OR = 1.460, 95%CI:1.012–2.107, *p* = 0.043), CD3 on EM CD4+ (OR = 1.245, 95%CI:1.000–1.550, *p* = 0.049), CD3 on TD CD8br (OR = 1.486, 95%CI:1.025–2.154, *p* = 0.037), CM CD4+ %T cell (OR = 1.762, 95%CI:1.019–3.046, *p* = 0.043), HVEM on naive CD4+ (OR = 1.451, 95%CI:1.104–1.907, *p* = 0.007). In the Monocyte group, CD16 on CD14+ CD16+ monocyte (OR = 1.285, 95%CI:1.018–1.621, *p* = 0.035), CD40 on CD14+ CD16+ monocyte (OR = 1.171, 95%CI:1.015–1.352, *p* = 0.030). In the Myeloid cell group, CD33dim HLA DR+ CD11b + %CD33dim HLA DR+ (OR = 1.262, 95%CI:1.020–1.561, *p* = 0.032). In TBNK group, FSC-A on NK (OR = 1.296, 95%CI:1.000–1.680, *p* = 0.049), HLA DR+ NK AC (OR = 1.568, 95%CI:1.100–2.237, *p* = 0.013), as shown in Figure 2 and Supplementary Table S1. Thirteen immune cell phenotypes as protective factors associated with GBS. For example, in the B cell group, CD19 on PB/PC (OR = 0.577, 95%CI:0.370–0.902, *p* = 0.016), IgD+ CD24− %lymphocyte (OR = 0.554, 95%CI:0.332–0.924, *p* = 0.024), CD8dim %leukocyte (OR = 0.621, 95%CI:0.405–0.953, *p* = 0.029), Transitional AC (OR = 0.704, 95%CI:0.524–0.946, *p* = 0.020). In the Treg cell group, CD127 on CD28− CD8br (OR = 0.727, 95%CI:0.541–0.976, *p* = 0.034), CD28+ CD45RA− CD8dim %T cell (OR = 0.863, 95%CI:0.771–0.966, *p* = 0.010), CD4 Treg AC (OR = 0.727, 95%CI:0.538–0.983, *p* = 0.038). In the cDC group, CD11c + monocyte %monocyte (OR = 0.704, 95%CI:0.514–0.966, *p* = 0.030), CD62L− myeloid DC AC (OR = 0.694, 95%CI:0.493–0.978, *p* = 0.037), CD86 on monocyte (OR = 0.737, 95%CI:0.544–0.999, *p* = 0.049). In the Monocyte group, CX3CR1 on CD14+ CD16− monocyte (OR = 0.717, 95%CI:0.548–0.9939, *p* = 0.016). In the Myeloid cell group, Mo MDSC AC (OR = 0.763, 95%CI:0.619–0.939, *p* = 0.011). In the TBNK group, CD45 on granulocyte (OR = 0.621, 95%CI:0.391–0.984, *p* = 0.042), as shown in Figure 3 and Supplementary Table S2.

### 3.2 Sensitivity analysis

**Figure 4:**
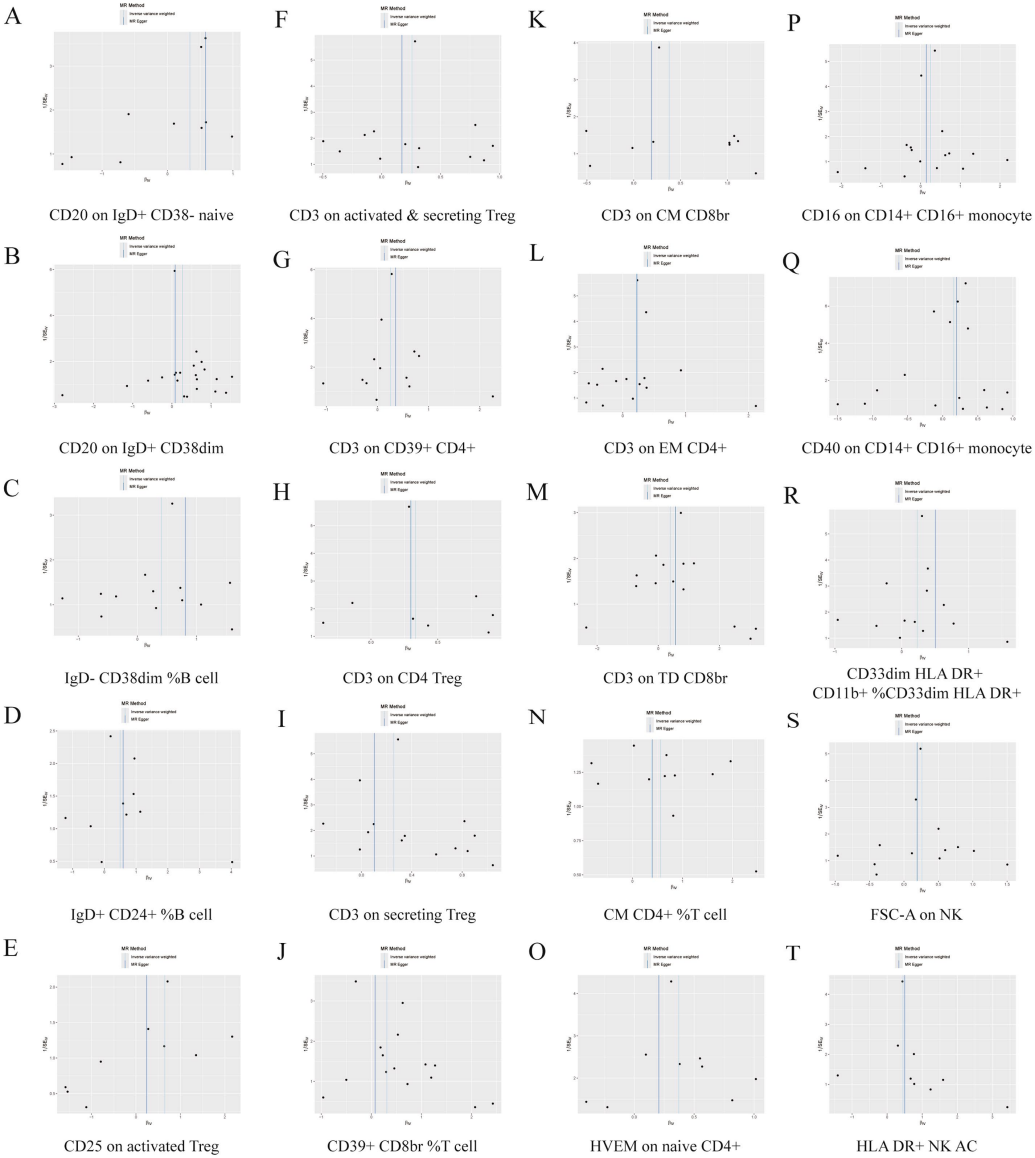
Funnel plot of 20 immune cell characteristics as risk factors for GBS. (A) CD20 on IgD+ CD38− naive. (B) CD20 on IgD+ CD38dim. (C) IgD− CD38dim %B cell. (D) IgD+ CD24+ %B cell. (E) CD25 on activated Treg. (F) CD3 on activated and secreting Treg. (G) CD3 on CD39+ CD4+. (H) CD3 on CD4 Treg. (I) CD3 on secreting Treg. (J) CD39+ CD8br %T cell. (K) CD3 on CM CD8br. (L) CD3 on EM CD4+. (M) CD3 on TD CD8br. (N) CM CD4+ %T cell. (O) HVEM on naive CD4+. (P) CD16 on CD14+ CD16+ monocyte. (Q) CD40 on CD14+ CD16+ monocyte. (R) CD33dim HLA DR+ CD11b + %CD33dim HLA DR+. (S) FSC-A on NK. (T) HLA DR+ NK AC.

**Figure 5:**
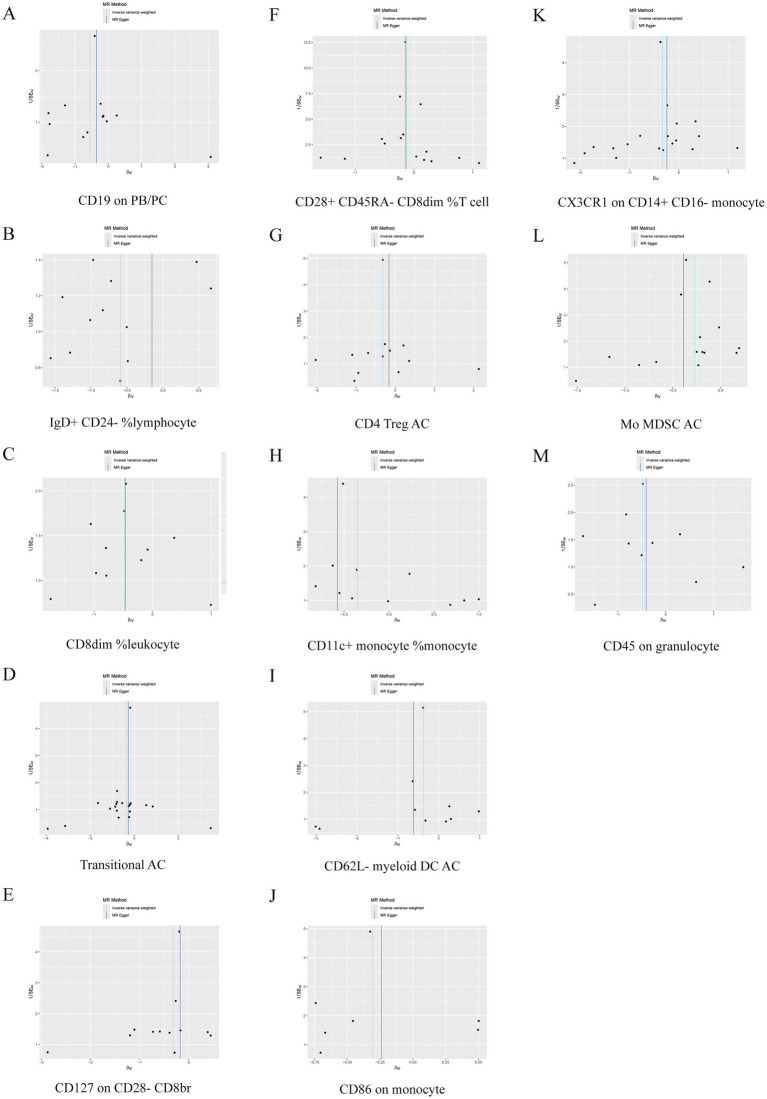
Funnel plot of 13 immune cell characteristics as protective factors for GBS. (A) CD19 on PB/PC. (B) IgD+ CD24− %lymphocyte. (C) CD8dim %leukocyte. (D) Transitional AC. (E) CD127 on CD28− CD8br. (F) CD28+ CD45RA− CD8dim %T cell. (G) CD4 Treg AC. (H) CD11c + monocyte %monocyte. (I) CD62L− myeloid DC AC. (J) CD86 on monocyte. (K) CX3CR1 on CD14+ CD16− monocyte. (L) Mo MDSC AC. (M) CD45 on granulocyte.

**Figure 6:**
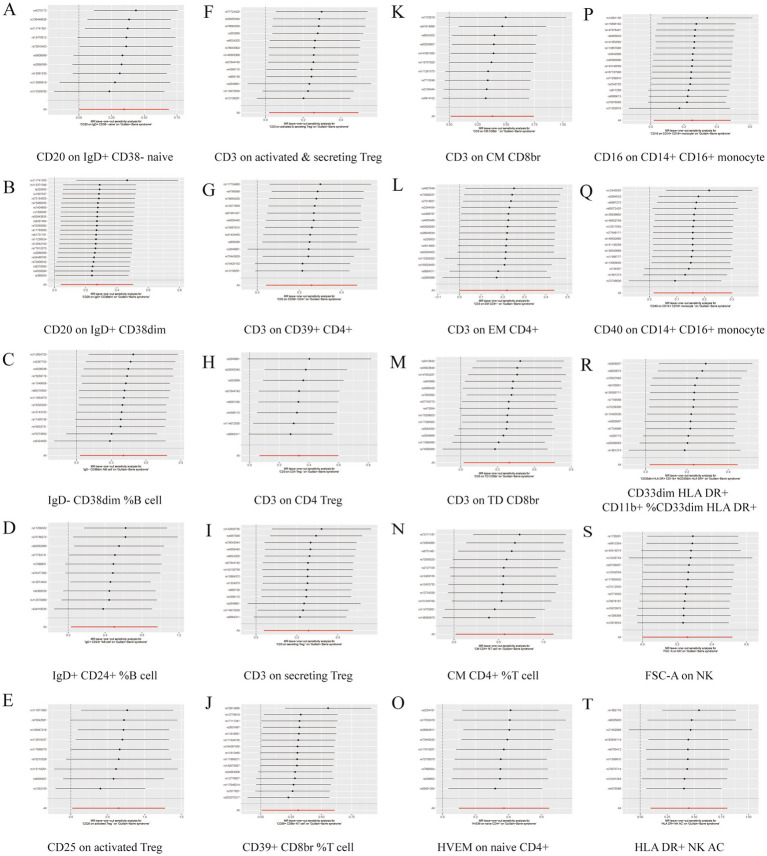
Leave one out forest plot of 20 immune cell characteristics as risk factors with GBS. (A) CD20 on IgD+ CD38− naive. (B) CD20 on IgD+ CD38dim. (C) IgD− CD38dim %B cell. (D) IgD+ CD24+ %B cell. (E) CD25 on activated Treg. (F) CD3 on activated and secreting Treg. (G) CD3 on CD39+ CD4+. (H) CD3 on CD4 Treg. (I) CD3 on secreting Treg. (J) CD39+ CD8br %T cell. (K) CD3 on CM CD8br. (L) CD3 on EM CD4+. (M) CD3 on TD CD8br. (N) CM CD4+ %T cell. (O) HVEM on naive CD4+. (P) CD16 on CD14+ CD16+ monocyte. (Q) CD40 on CD14+ CD16+ monocyte. (R) CD33dim HLA DR+ CD11b + %CD33dim HLA DR+. (S) FSC-A on NK. (T) HLA DR+ NK AC.

**Figure 7:**
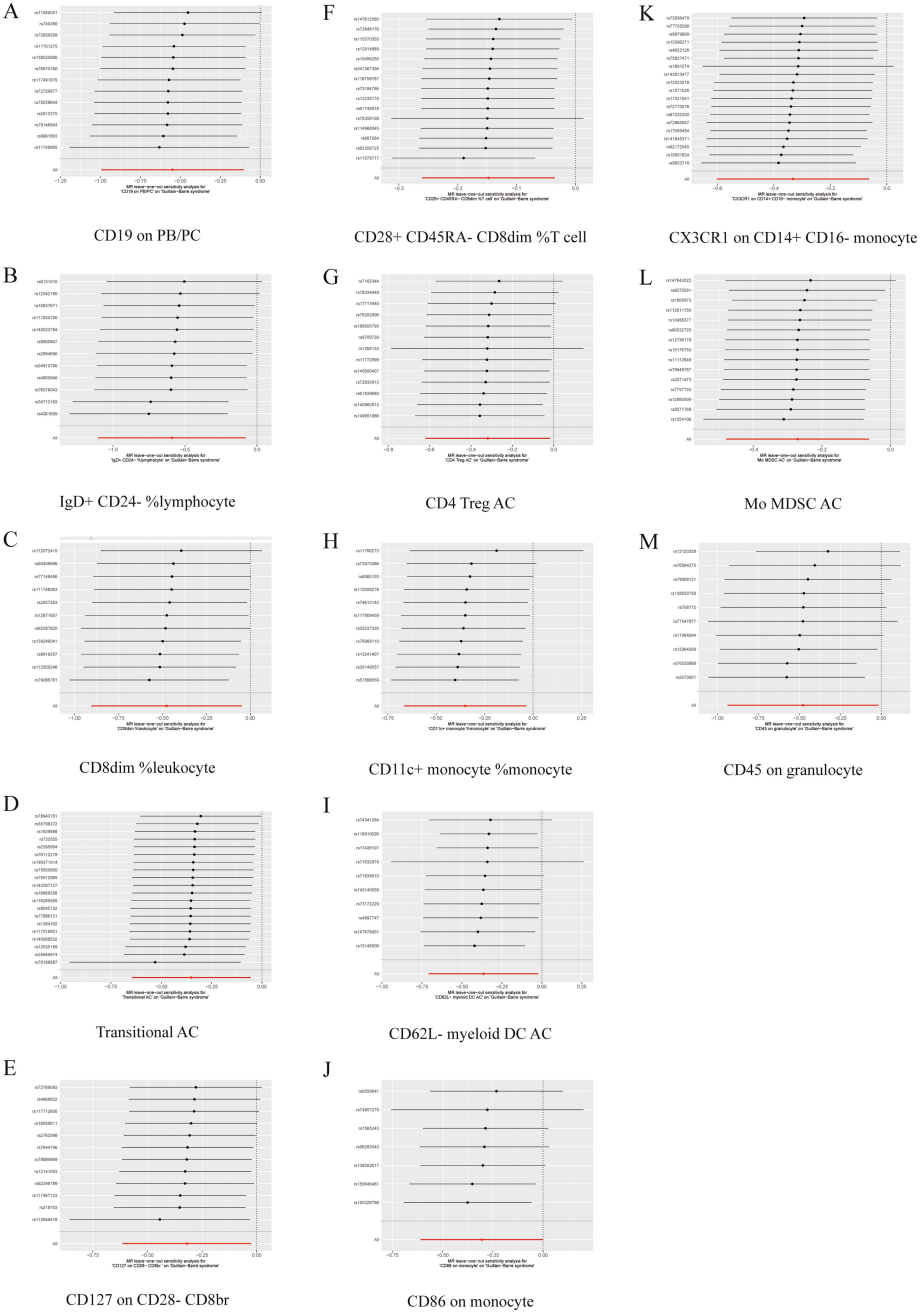
Leave one out forest plot of 13 immune cell characteristics as protective factors against GBS. (A) CD19 on PB/PC. (B) IgD+ CD24− %lymphocyte. (C) CD8dim %leukocyte. (D) Transitional AC. (E) CD127 on CD28− CD8br. (F) CD28+ CD45RA− CD8dim %T cell. (G) CD4 Treg AC. (H) CD11c + monocyte %monocyte. (I) CD62L− myeloid DC AC. (J) CD86 on monocyte. (K) CX3CR1 on CD14+ CD16− monocyte. (L) Mo MDSC AC. (M) CD45 on granulocyte.

Further sensitivity analyses of the results of the significant causal relationship between immune cell phenotype and GBS using Cochran’s *Q* test showed that Q_pval was >0.05 in all cases and there was no significant heterogeneity, no outliers were found in the MR-PRESSO results, and there was no level of The intersection of the MR-Egger regression pleiotropy (pval >0.05) as shown in Figures 4, 5 and Supplementary Table S3. Leave-one-out analyses provide some evidence of the robustness of the results of this part of the study, as shown in Figures 6, 7.

### 3.3 Inverse MR analysis results

To investigate the causal relationship between GBS and immune phenotypes, we used inverse MR to study the effect of GBS on immune phenotype cells. The results showed that there was no causal relationship between GBS and any of the 33 immune cells mentioned above.

## 4 Discussion

The GBS usually develops after infection and affects the peripheral nervous system, resulting in muscle weakness and sensory abnormalities, with the condition progressing gradually from mild muscle weakness to severe generalized paralysis. In recent years, the scientific hypothesis that “the immune system attacks the nervous system to cause disease” has gradually come to the forefront of researchers’ minds as studies on the pathogenesis of GBS continue to deepen. This study analyzed the potential causal relationship between 731 immune cell phenotypic markers and GBS using MR methods based on a large amount of publicly available genetic data. The results of the analysis showed that a total of 33 immune cell phenotypes were included in this study, 20 immune cell phenotype markers were considered as risk factors for the development of GBS and 13 immune cell phenotype markers were considered as protective factors for the development of GBS. There were 9 cases in the Treg cell group, 7 cases in the B cell group, 5 cases in the Maturation stages of T cell group, 4 cases in the TBNK group, 3 cases in the cDC cell group, 3 cases in the Monocyte group and 2 cases in the Myeloid cell group.

Treg cells play an important role in maintaining immune homeostasis and suppressing inflammation, and a reduction in their numbers or dysfunction may be importantly linked to the pathogenesis of GBS. Patients with AMAN and AIDP, a common subtype of GBS, have significantly fewer peripheral Tregs in the acute phase of the disease (16). Immunosuppressive subpopulations of CD4+ T helper cells reduce autoimmune and inflammatory responses and are widely used in the treatment of neurological disorders such as GBS (17). It has been shown that patients with GBS treated with lymphoid progenitor exchange (LPE) have a significant decrease in the percentage of Th1 and Th17 cells and an increase in Th2 and Treg cells in the peripheral blood among the CD4+ T-lymphocyte subsets (18). It follows that the role of Th17 cells in GBS should not be overlooked, and these cells promote GBS by mediating inflammatory and autoimmune responses (19).The results of another similar study suggest that during the acute phase of the clinical course of GBS, although there is a decline in the number and proportion of CD4 + CD25+ T cells, this decline is reversible, suggesting that the number and function of Treg cells may be only temporarily suppressed rather than permanently impaired (20). IVIG promotes proliferation of CD4 + CD25 + Foxp3 + and other Treg cells and secretion of anti-inflammatory cytokines IL-10 and TGF-β1 in GBS patients (21). Experimental autoimmune neuritis (EAN) is an animal model of AIDP that induces T-cell-mediated neuritis via myelin epitopes P0 or P2 as the primary antigen (22). Pathologically, EAN is characterized by rupture of the blood-nerve barrier and accumulation of autoreactive T cells and macrophages in the peripheral nervous system through chemotaxis and demyelination (23). *In vitro* activation of anti-CD3 and anti-CD28 antibodies allows IFN-*γ* to generate CD4 + CD25+ regulatory T cells (iTregs) from CD4 + CD25− T cells in GBS patients (24). The above further corroborates the association of Treg cell subtypes such as CD25 on activated Treg, CD3 on activated and secreting Treg, CD3 on CD4 Treg and GBS in the results.

B cells are involved in GBS pathogenesis through autoantibody production, antigen presentation, and inflammatory regulation. Flow cytometry results showed that the percentage of memory B cells was significantly higher in GBS patients than in healthy controls. Correlation analyses showed that an increase in the percentage of memory B cells was positively correlated with the clinical severity of GBS patients (25). This was similarly corroborated in another study, suggesting that peripheral blood DN (IgD− CD27−) B-cell values were significantly elevated (26). The decrease in the percentage of circulating memory B cells after administration of immunotherapies such as PE and IVIG to GBS patients suggests that the reduction of memory B cells is involved in the recovery process of the disease or is one of the key factors for clinical improvement. For example, the percentage of CD4+/CD8+ T cells and the percentage of CD4 + CD45RA + T cells were increased, the percentage of CD8 + T cells and CD4 + CD45RO + T cells were significantly decreased, and the number of CD19 + B cells was reduced after IVIG treatment in GBS patients (27). It also promotes the differentiation of CD40-activated B cells into plasmoblasts and accelerates immunoglobulin synthesis and secretion (28). Large numbers of plasmoblasts may be a potential biomarker for rapid clinical recovery. CD19 can be used as a B-cell target to treat autoimmune neuropathies (29). It has also been shown that an anti-CD20 monoclonal antibody (rituximab) may be a potential target for the treatment of GBS (30). The results of the present study are broadly similar to the above, CD19 on PB/PC, CD20 on IgD+ CD38− naive, CD20 on IgD+ CD38dim are associated with GBS.

Myeloid cells are a group of cells that play an important role in the immune system and include mainly granulocytes, monocytes, macrophages, dendritic cells and mast cells. These cells are derived from hematopoietic stem cells in the bone marrow and are formed through a series of differentiation processes that are also closely linked to the pathogenesis of GBS. Antigen-presenting cell activity of myeloid dendritic cells may contribute to the maintenance of T-cell activation in GBS, with increased numbers of CD11c(+) myeloid DCs versus CD123(+) plasma cell-like DCs in patients with GBS before treatment with high-dose IVIG (31). Another study showed that plasma cell-like DCs were significantly elevated in the acute phase of GBS, and their levels were positively correlated with the severity of disease in GBS patients, and the expression of TLR9 and surface co-stimulatory molecules were significantly elevated in plasma cell-like DCs, suggesting that plasma cell-like DCs are involved in the pathogenesis of GBS (32). Atorvastatin-modified DCs can be induced into tolerogenic DCs, which improve the symptoms of EAN in rats by down-regulating Th1/Th17 levels and increasing the number of Treg and NKR-P1+ cells (33). Although DCs-based immunotherapy is still at the stage of animal experiments, the results of existing studies suggest that DCs have a promising application in the clinical treatment of GBS (34). CD16 + 56, CD4+ and CD8+ levels were lower and IgG levels were higher in children with GBS spectrum disease variant than in the control group, suggesting that both cellular and humoral immune functions were disturbed in children with GBS spectrum disease variant and were involved in the development of the disease (35). Single-cell RNA sequencing of peripheral blood mononuclear cells (PBMC) from patients with GBS revealed a new clonally-expanded CD14+ CD163+ monocyte subset in the peripheral blood of patients with AIDP and it was enriched for cellular responses to IL1 and chemokine signaling pathways (36). In addition, the researchers found that the neutrophil/lymphocyte ratio (NLR) may be an independent risk factor for GBS and a predictor of severe dysfunction, severe frailty and short-term prognosis (37). Macrophages can be divided into two main phenotypes, pro-inflammatory macrophages (M1) and anti-inflammatory macrophages (M2), which play a decisive role in the initiation and development of GBS. Macrophages may induce inflammatory or anti-inflammatory effects in M1 and M2 by secreting pro- or anti-inflammatory cytokines (TNF-*α*, IL-12, IL-10, etc.), and induce activation of T cells to mediate immune responses or to promote GBS disease recovery. Currently, the role of macrophages in, e.g., GBS cannot be explained simply by the M1–M2 dichotomy, and how macrophages are involved in degeneration and regeneration of the peripheral nervous system, how macrophage polarization can be shifted toward the M2 phenotype, and how to improve the outcome of GBS need to be explored in further studies (38). The relevant results of the present study on myeloid cell categories are in general agreement with the literature, suggesting that the phenotypes: CD11c + monocyte %monocyte, CX3CR1 on CD14+ CD16− monocyte, CD16 on CD14+ CD16+ monocyte, etc., may be closely related to the GBS.

In addition, reverse MR analyses were performed for further validation of the positive results of this study. The results showed that there was no causal relationship between GBS and any of the 33 immune cells. The reason for this may be that genetic variants predominantly precede disease, and the sequence of the two cannot usually be reversed.

In summary, this work differs from traditional observational studies that address the relationship between one or more immune cells and GBS. It used MR analysis with SNPs as IVs to investigate causal associations between 731 immune cell phenotypes and GBS, reducing confounding variables, reverse causation, and other factors interfering with the results. The results suggest a causal link between 33 immune cell manifestations and GBS. They may play a defensive or pathogenic role by activating different immune functions, and the B-cell and Treg cell groups dominate the exposure factors for GBS. These findings provide a theoretical basis for the development of early detection reagents and late treatment strategies.

In this study, Mendelian randomization (MR) analysis was used to investigate the relationship between GBS and immune cell phenotypes. Although this analysis provides important information on causality, the following limitations exist. For example, the data on 731 immune cell phenotypes and GBS were derived from studies of European populations, and the presence of similar genetic variants in other populations needs to be further explored. If the selected SNPs affect multiple biological pathways, their effects on immune cell function may not be exclusive, potentially introducing a horizontal confounding bias, which in turn affects the accuracy of the MR analysis. Synergistic effects between different immune cell phenotypes were not discussed in this study, and their impact on the findings cannot be ignored. Due to these limitations, future clinical studies with more ethnic groups, more comprehensive genotypic data, and more scientific methods of statistical analyses are needed to enhance the ability of MR in explaining the relationship between GBS and immune cell phenotypes.

## Data Availability

The original contributions presented in the study are included in the article/Supplementary material, further inquiries can be directed to the corresponding author.
