# Supplementary material for: Causal relationship between immune cells and Guillain-Barré syndrome: a Mendelian randomization study
Source: Front Neurol. 2024 Nov 12;15:1446472. doi: 10.3389/fneur.2024.1446472 (PMC11588641; doi:10.3389/fneur.2024.1446472)
Supplement: Supplementary file 1 [file Table_1.docx]

Table 1 Summary information on GWAS data

| **GWAS Data** | **Sample Size** | | **Population** | **Data sources** | **SNP(n)** | **Year** |
| --- | --- | --- | --- | --- | --- | --- |
| immune cells | | 3757 | European | ebi-a-GCST0001391-  ebi-a-GCST0002121 | About 2,000,000 | 2020 |
| Guillain-Barre syndrome | | 215931 | European | finn-b-G6_GUILBAR | 16,380,463 | 2021 |
